# Mechanical Thrombectomy in the Management of Acute Ischemic Stroke Secondary to Calcified Cerebral Emboli: a Systematic Review

**DOI:** 10.1007/s00062-025-01611-7

**Published:** 2026-01-28

**Authors:** Nicholas V. Pavic, Shane Zhang, Alexander G. Maloof, Mobin Aber, Christie Wu, Stephen D. Bacchi, Vinicius Carraro Do Nascimento, Rudy Goh

**Affiliations:** 1 Blacktown Hospital, Blacktown, Australia; 2 Western Sydney University, Blacktown, Australia; 3 Royal North Shore Hospital, St Leonards, Australia; 4 Prince of Wales Hospital, Randwick, Australia; 5 Adelaide University, Adelaide, Australia; 6 Gold Coast University Hospital, Southport, Australia

**Keywords:** Endovascular recanalisation, Clot retrieval, Calcific embolus, Mechanical thrombectomy

## Abstract

**Purpose:**

Mechanical thrombectomy (MT), in combination with intravenous thrombolysis, have been widely accepted as effective therapies for acute ischemic stroke (AIS) secondary to large vessel occlusion. However, the presence of calcified cerebral emboli (CCE) has been associated with worse angiographic and clinical outcomes. Therefore, this systematic review aimed to evaluate the efficacy and safety of MT in the management of AIS secondary to CCE.

**Methods:**

A systematic review was registered and conducted in accordance with the Preferred Reporting Items for Systematic Reviews and Meta-Analyses guidelines (CRD420251031539). PubMed, Ovid Medline, SCOPUS, The Cochrane Library and EMBASE were searched for publications until 13th April 2025.

**Results:**

The search yielded 2102 publications. 14 studies met inclusion criteria (160 patients with AIS due to CCE). Two multi-centre studies reported recanalisation rates of only 57.5% and 57% respectively across 75 participants. Only 11.1–28.0% of patients in the included case series and cohort studies had a modified rankin score 2 at 3 months, suggesting that most patients did not regain functional independence following MT for CCE. MT for CCE was also associated with a 3-month mortality rate of 0–62.5%. Haemorrhagic complications were the most common adverse effect associated with MT.

**Conclusion:**

The available evidence from 14 studies elucidated poor recanalisation rates and functional outcomes when MT was used in the management of AIS secondary to CCE. However, the available evidence is primarily low-level evidence from observational studies, hence the conclusions should be interpreted with caution.

**Supplementary Information:**

The online version of this article (10.1007/s00062-025-01611-7) contains supplementary material, which is available to authorized users.

## Introduction

Mechanical thrombectomy (MT), in combination with intravenous thrombolysis, have been widely accepted as effective therapies for acute ischemic stroke (AIS) secondary to large vessel occlusion (LVO) [1, 2]. MT is performed using an aspiration catheter or stent-retriever, with the aim of attaining near complete or complete recanalisation of the ischemic territory [3]. Although new generation devices are associated with higher recanalisation rates, MT fails to achieve target vessel recanalisation, as defined by an extended thrombolysis in cerebral infarction (eTICI) score of 1 to 2a, in up to 15 to 20% of cases [4]. Thrombectomy failure may be influenced by several factors, including underlying intracranial atherosclerosis, angulation of the middle cerebral artery, and thrombus composition [5–7].

Among patients with AIS, calcified cerebral emboli (CCE) have an estimated prevalence of between 2.7 and 5.9%, and are associated with worse angiographic and clinical outcomes [4, 8]. CCE are typically identified on CT imaging as round and hyperdense with a higher attenuation (~160 Hounsfield Units) compared to regular thrombi [9]. CCE may occur spontaneously in the setting of calcific aortic stenosis or vascular calcification, or be iatrogenic following percutaneous cardiac interventions or surgical carotid endarterectomy [8].

The management of AIS secondary to CCE remains a significant challenge. As CCE is a rare cause of AIS, therapeutic experience is scarce [10]. Medical management alone is seldom effective, as thrombolysis targets the fibrin component of the embolus and does not impact calcium [11]. Moreover, calcified clots contain a significant amount of calcium phosphate, which may influence their mechanical properties. The hard, irregular and sharp-edged nature of the CCE increases the difficulty of thrombectomy, and heightens the risk of damage to the vessel wall [11]. In this context, two recent retrospective multi-centre studies concluded that the post-thrombectomy recanalisation rate of AIS secondary to CCE was only 57% and 57.5% respectively [4, 12].

Thus, this systematic review aimed to evaluate the current available evidence regarding the efficacy and safety of MT in the management of patients with AIS secondary to CCE.

## Materials and Method

This systematic review was registered with PROSPERO (CRD420251031539) and is reported in accordance with the Preferred Reporting Items for Systematic Reviews and Meta-Analysis (PRISMA) guidelines (see online supplemental information 1) [13].

### Study Design

The population, intervention, comparator and outcome (PICO) framework was used to form the research question. The population included adults with clinically diagnosed AIS and imaging findings suggestive of CCE. The intervention was MT. The comparator group consisted of those receiving either thrombolysis without thrombectomy or no hyperacute stroke treatment. The primary outcome was endovascular recanalisation rates, as measured by the extended thrombolysis in cerebral infarction (eTICI) grading system. Successful recanalisation was defined as an eTICI score greater or equal to 2b. Secondary outcomes included the National Institutes of Health Stroke Scale/Score (NIHSS) at various time points (e.g. 24 h, 2 weeks, 90 days), functional outcomes as assessed by the Modified Rankin Scale (mRS), all-cause mortality, and incidence of adverse events.

### Data Sources and Search Strategy

On the 13th April 2025 a thorough search was performed using PubMed, OVID Medline, SCOPUS, The Cochrane library and EMBASE. There was no restriction on publication period. The search terms applied were as follows: (Calcif*) AND (embol*) AND (thrombectomy OR endovascular OR recanalisation OR retrieval). The search strings are available in the supplementary information (see online supplemental information 2). We manually searched the reference list of included studies and previous reviews on this subject. We aimed to include both published and unpublished studies.

### Study Eligibility

Studies were eligible for inclusion if they evaluated the efficacy and safety of MT in the management of AIS secondary to CCE. We sought to include case reports, case series, case-control, cohort and randomised controlled studies.

The exclusion criteria included (a) previous reviews, conference abstracts, editorials or letters, (b) animal-based studies, (c) pre-clinical trials, (d) studies that assessed thrombolysis without MT or surgical embolectomy without a prior attempt of MT, (e) no available English translation or (f) studies that included paediatric or neonatal strokes.

### Data Extraction

We used the Covidence systematic review software (Veritas Health Innovation, Melbourne, Australia) to screen studies according to the inclusion and exclusion criteria. Two reviewers (MA and CW) independently screened titles and abstracts while a third reviewer (NP) resolved disputes. This process was followed by a full-text screen.

The following data was extracted from the included studies; first author (year), study design, dataset characteristics, MT technique, administration of thrombolysis, whether rescue therapies were required, follow-up time, and primary and secondary outcomes (refer to Table 1 and 2).

**Table 1:** Characteristics of studies included in review

First author (year) Region	Study design	Dataset characteristics	Mechanical thrombectomy technique	Thrombolysis administered (yes/no)	Rescue therapies attempted (yes/no)	Follow-up time	Primary outcome	Secondary outcome
Schirmer [16] USA	Case report	1 patient (75 year old female) with CCE-related AIS secondary to cardiac catheterization who underwent thrombectomy	Alligator retrieval device and Merci Retrieval System	No	Yes, coronary balloon-mounted stent followed by Taxus stent	Postprocedural CT, follow up CT angiogram at 8 months, MRI at 2 days, follow up MR angiogram at 1 year	Not applicable	Not applicable
Koh [17] Korea	Retrospective case series	5 patients with AIS secondary to calcific emboli	Continuous manual aspiration was performed using a reperfusion penumbra catheter	Yes (4 of 5 patients received IV rTPA)	No	NIHSS measured at discharge, mRS at 3 months post stroke	Recanalisation rate	Functional outcomes (mRS ≤ 2)
Dobrocky [18] Switzerland	Retrospective case series	8 patients (mean age of 80) with acute ischemic stroke due to calcified intracranial embolus who underwent thrombectomy	Stent retriever	Yes (2 of 8 patients received IV tPA)	No	3 months	Recanalisation rate (TICI 2 b–3), functional outcome (mRS 0–2 at 3 months) and mortality rate	Complications after thrombectomy and presumed source of emboli
Kwak & Park [19] South Korea	Case report	1 patient (82 year old female) with AIS due to CCE in the left MCA who underwent MT	Manual aspiration thrombectomy (MAT) and	Yes	Yes	Post-thrombectomy DSA	Not applicable	Not applicable
			Embolus Retriever with Interlinked Cage (ERIC) device		Thrombectomy with ERIC device	3 months follow up which assessed mRS		
Murakami ; [20]) Japan	Case report	1 patient (73 year old male) with CCE-related AIS in the right MCA and PCA after TAVI and PCI, who underwent endovascular thrombectomy	Stent retriever	No	No	Post-thrombectomy cerebral angiography 6 days and 30 days after thrombectomy	Not applicable	Not applicable
Maurer [12] Germany, Switzerland and France	Retrospective multicenter study	40 patients (with a mean age of 78 ± 9.6) with AIS due to CCE who underwent MT	A distal aspiration catheter alone was used in 2 patients (5%), while a stent retriever alone was utilised in 2 patients (5%). 36 patients (90%) used a combination of both	Yes. 19 of 40 (47.5%) patients received IV thrombolysis	Yes. Intracranial stent was performed in 2 patients (5%)	24 h (ASPECTS) 90 days	Recanalisation rate (mTICI), good functional outcome (mRS ≤ 2) and CCE features leading to low recanalisation rate	Procedural complications, reasons resulting in high 90-day mortality rate and use of intracranial stents after failed thrombectomy
Bres Bullrich [11] Canada	Case report	1 patient (78 year old male) with AIS secondary to CCE who underwent thrombectomy	Direct Aspiration First Pass Technique (ADAPT)	No	No	24 h (Head CT), 11 days (brain MRI)	Not applicable	Not applicable
Bruggeman [21] Netherlands	Prospective cohort study	55 patients with intracranial anterior circulation occlusion due to CCE (median age of 76), compared to 3022 patients without CCE	Stent retriever (*n* = 30) and Aspiration (*n* = 14)	Yes (49 of 55 patients received thrombolysis)	Balloon guided catheter (*n* = 25)	3 months	Ordinal mRS at 90 days	eTICI of 2 b or more; median NIHSS improvement at 24–48 h
Grand [4] France	Multicentric retrospective observational nationwide study	35 patients, with an average age of 76, who underwent thrombectomy due to acute ischemic stroke as a result of CCE causing a proximal large vessel occlusion, across 37 stroke centres	Stent retriever ± aspiration (*n* = 29, 83%)	Yes (11 patients received thrombolysis (31%))	Balloon guided catheter (*n* = 16) (46%)	3 months	Recanalisation rates (mTICI > 2 B) and good clinical outcome (mRS < 2)	Median NIHSS at discharge and complications were analysed
			Contact aspiration alone (*n* = 6, 17%)					
Mosqueira [22] Spain	Retrospective case series	9 patients with a mean age of 79.1 years, who developed stroke due to CCE. Only 1 patient underwent thrombectomy	Aspiration catheter	No	No	3 months	Not applicable	Not applicable
Vishwanath [23] USA	Case report	66 year old female with a left MCA CCE who underwent thrombectomy	Not mentioned	No	No	No follow-up post discharge	Not applicable	Not applicable
Azad [24] USA	Case report	Pregnant patient (early 30 s) presenting with acute stroke due to CCE (with a pelvic phlebolith origin) who underwent thrombectomy	Balloon sweeps and simultaneous aspiration (unsuccessful after 5 attempts)	No	Yes, Surgical endarterectomy and embolectomy	4 months	Not applicable	Not applicable
Yokochi [25] Japan	Case report	71 year old patient presenting with AIS secondary to CCE who underwent thrombectomy	Stent retriever + aspiration catheter	No	No	3 months mRS and 6 months review for stroke recurrence	Not applicable	Not applicable
Chiaroni [26] France	Case report	64 year old patient presenting with AIS due to ICA thrombus which later embolised, who underwent thrombectomy followed by stenting	Stent retriever + aspiration catheter	No	Yes, intracranial stenting	Non-contrast CT and CT angiogram (following day), discharged 12 days after onset	Not applicable	Not applicable

Abbreviations: *CCE* Calcified cerebral emboli, *MT* mechanical thrombectomy, *AIS* acute ischemic stroke, *TPa* tissue-type plasminogen activator, *mRS* modified Rankin Scale,* NIHSS* National Institutes of Health Stroke Scale, *TICI scale* thrombolysis in cerebral infarction scale, *ICA* internal carotid artery, *MCA* middle cerebral artery, *MAT* mechanical aspiration thrombectomy

**Table 2:** Key results of studies included in review

First author (year)	Endovascular recanalisation rates	National Institutes of Health Stroke Scale/Score (NIHSS) at various time points	Modified Rankin Score (mRS)	Mortality rate	Adverse effects	Other- relevant findings
Schirmer [16]	Following rescue therapy, CT head showed the impingement of CCE onto the proximal stent, indicating successful retrieval. MR angiography revealed persistent flow restoration of left MCA	Not Assessed	Not assessed	Not assessed	No adverse effects reported	N/A
Koh [17]	MAT did not remove calcified emboli in all 5 patients	Initial median NIHSS on admission was 9. After MT, the median NIHSS at discharge was 4	Initial median mRS on admission was 4. Median mRS at 3 month follow-up was 3	No hospital or 3‑month mortality occurred	No adverse effects reported	N/A
Dobrocky [18]	1 of 8 patients (12.5%) achieved successful recanalisation after thrombectomy (TICI 2 b–3)	Initial mean NIHSS on admission was 13.5 (Interquartile range 10.25–17.25). NIHSS after thrombectomy was not assessed	mRS at 3 months were as follows: 1 patient (12.5%) had a score of 0–2, 1 patient (12.5%) scored 5 and was severely disabled, 5 patients (62.5%) scored 6 and died. 1 patient was not followed up	3‑month mortality rate of 62.5% (5 of 8 patients) by 3 month follow-up	Detachment of stent retriever without removal (*n* = 1), extensive subarachnoid haemorrhage (*n* = 1)	Presumed sources of emboli were carotid atherosclerotic plaques (62.5%), TAVI (12.5%) and undetermined (25%)
Kwak & Park [19]	MAT failed to remove CCE. MT using the ERIC device was attempted and complete recanalisation was achieved at the left M1 branch of MCA	NIHSS on admission was 13 and improved to 4 after thrombectomy	mRS score of 1 at 3 months	No mortality at 3 months	No adverse effects reported	N/A
Murakami [20]	Patient achieved complete recanalisation in the right MCA (TICI 3). recanalisation at the right PCA was not attained as it was too small	Not assessed	mRS score of 3 at 30 days	No mortality at 30 days	Haemorrhage in the right MCA was shown on CT a day after thrombectomy	Histopathological features of the emboli were a mixture of red and white thrombi, calcium fragments, inflammatory cells and cholesterol deposits
Maurer [12]	23 of 40 patients (57.5%) achieved mTICI ≥ 2 b	Median NIHSS on admission was 14 (IQR 11–20). NIHSS was not assessed post-thrombectomy	mRS at 90 days was assessed in 34 patients. Functional independence of mRS 0–2 was achieved in 9 of 34 patients (26.5%) while mRS ≥ 3 was achieved in 25 of 34 patients (73.5%)	The 90-day mortality rate was 55.9% (19/34 patients)	4 of 40 patients had post-thrombectomy complications. Two patients with symptomatic haemorrhage, two patients with vessel perforation	Median ASPECTS score on admission was 9 (IQR 7–9) and transitioned to a median of 5, 24 h after thrombectomy
	4 of 40 patients (10%) achieved mTICI 2 a					
	13 of 40 patients (32.5%) achieved mTICI 0–1					
Bres Bullrich [11]	1 patient achieved full recanalisation post MT (TICI 3)	NIHSS on admission was 4, which then progressed to 7 before thrombectomy	mRS score of 0 at discharge (48 h after stroke onset)	Not assessed	No adverse effects reported	N/A
		NIHSS of 0 at discharge				
Bruggeman [21]	Successful recanalisation (eTICI of 2 b or more) reported in 44% of CCE patients compared to 63% of non-CCE patients (OR 0.52, 95% CI 0.28–0.95, and adjusted OR [aOR] 0.52, 95% CI 0.28–0.97)	CCE patients achieved a median improvement of 2 points on the NIHSS 24–48 h post EVT, while non-CCE patients achieved a median improvement of 4 points (*p* = 0.008)	After 3 months, 29% (15/51 of CCE patients achieved functional independence (mRS score 0–2), versus 41% (*n* = 1159/2823) of non-CCE patients (*p* = 0.09). After propensity matching, the presence of CCE was not significantly associated with worse functional outcome (ucOR 0.90, 95% CI 0.39–2.09, and acOR 0.88, 95% CI 0.33–2.33) Note 4 patients of the original *n* = 55 sample were lost to follow-up	90-days was reported in 35% (18/51) of CCE patients and 29% (808 of 2823) of non-CCE patients. CCE presence was not significantly associated with mortality (OR 1.25, 95% CI 0.71‑2.21, and aOR 1.16, 95% CI 0.64–2.12)	Symptomatic intracranial haemorrhage occurred in 8 CCE patients (15%), compared to 171 of 3022 non-CCE patients (6%; *p* = 0.01)	Three thrombi of CCE patients were analysed. Calcifications were only found in 1 thrombus
Grand [4]	20 patients (57%) achieved an mTICI of 2 B–3 post thrombectomy	Baseline median NIHSS was 15 (Interquartile range 9–18). Median NIHSS at discharge was 8 (IQR 2–15)	Baseline median mRS was 0 (IQR 0–1), and 8 patients (28%) achieved an mRS of 0–2 at the 3 month follow up	Not assessed	Total of 8 (22.9%) procedure related complications, 4 major (symptomatic intracranial haemorrhage), 4 minor (asymptomatic intracranial haemorrhage)	Age influenced recanalisation outcomes (*p* = 0.047). Number of thrombectomy device passes correlated with poor outcomes (*p* < 0.05)
Mosqueira [22]	1 patient underwent MT which was unsuccessful	Median NIHSS at admission was 8 (Q1–Q3: 5–17). NIHSS at follow-up was not mentioned	Median mRS at 3 month follow up was 4 (Q1–Q3: 2–6), and 4 (44.4%) patients had an mRS ≥ 4.1 (11.1%) patient achieved an mRS ≤ 2	Not assessed	No adverse effects reported	N/A
Vishwanath [23]	TICI 2B post recanalisation	Not assessed	Not assessed	Not assessed	No adverse effects reported	N/A
Azad [24]	Unsuccessful thrombectomy, TICI 3 following surgical embolectomy	Not assessed	mRS of 1 at 4 month follow up	Not assessed	No adverse effects reported	N/A
Yokochi [25]	TICI 3 post recanalisation	NIHSS on presentation was 10, and was not reported in the follow up results	mRS on the day of discharge (25 days post onset) was 1, and then 0 in the 90 day follow up	Not assessed	No adverse effects reported	N/A
Chiaroni [26]	mTICI 3 post recanalisation	Initial NIHSS was 23. No reported follow-up NIHSS, however (persistent severe aphasia was reported on discharge)	Not reported	Not assessed	Embolisation of the thrombus in the ICA to the MCA during thrombectomy	No haemorrhagic complications

Abbreviations: *CCE* Calcified cerebral emboli, *MT* mechanical thrombectomy, *AIS* acute ischemic stroke, *TPa* tissue-type plasminogen activator, *mRS* modified Rankin Scale, *NIHSS* National Institutes of Health Stroke Scale, *TICI scale* thrombolysis in cerebral infarction scale, *mTICI scale* modified thrombolysis in cerebral infarction scale, *eTICI scale* extended thrombolysis in cerebral infarction scale, *ICA* internal carotid artery, *MCA* middle cerebral artery

### Quality Assessment

The quality of included studies was assessed independently by two reviewers (MA and CW) (see online supplemental information 3). The Newcastle Ottawa Quality Assessment scale was used to evaluate bias in case control and cohort studies [14]. The Murad Tool was employed to analyse the risk of bias in case studies and case series [15]. Disagreement was resolved via consensus. Due to the paucity of included studies, publication bias was not assessed.

### Ethical Approval

As this study synthesises pre-existing published data on anonymised human subjects, ethics approval was not sought.

## Results

**Fig. 1:**
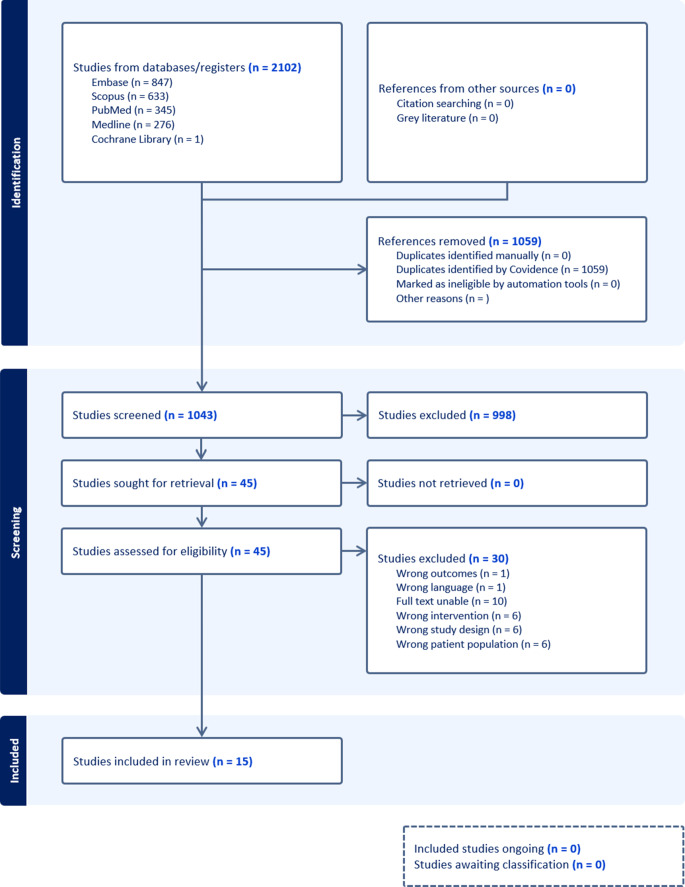
PRISMA diagram reporting the search strategy and the application of the inclusion and exclusion criteria for articles that assessed the utility of MT of CCE in AIS

The initial search of the five databases returned 2102 publications (Fig. 1). After removing 1059 duplicates through automated methods, 1043 publications were included for title and abstract screening. From these, 45 studies were selected for full-text review. Studies were excluded for several reasons, including; wrong outcomes (*n* = 1), wrong language (*n* = 1), wrong intervention (*n* = 6), wrong study design (*n* = 16) and wrong patient population (*n* = 7). In total, 14 studies met the inclusion criteria (refer to Table 1 and 2).

### Endovascular Recanalisation Rates

The eTICI grading scale is a value between 0 and 3, with an eTICI score of ≥ 2b usually indicating successful cerebral recanalisation [27]. Six studies used the original TICI grading scale [11, 18, 20, 23–25], three studies used the modified TICI (mTICI) scale [4, 12, 26], and one study used the extended TICI (eTICI) scale [21]. The heterogeneity in grading scales limits the potential comparability of recanalisation results across the included studies. In this context, recanalisation rates are presented consistent with the grading scale used in each individual study. The proportion of patients with TICI/mTICI/eTICI score ≥ 2b ranged from 0% to 57.5% across the included case series and cohort studies. The highest rates of recanalisation (57.0% and 57.5%) were observed in the two multicentre retrospective studies [4, 12]. One study compared recanalisation rates between AIS patients with and without CCE, reporting that 44% of patients with CCE had an eTICI score ≥ 2b following MT, compared to 63% in the non-CCE group [21]. Four of the eight individual case reports [11, 20, 23, 25] demonstrated a TICI score of ≥ 2b. Overall, most studies reported low rates of recanalisation.

### Clinical Assessment Scores at Follow-up

A mRS score of 0 to 2 at 90 days indicates good functional recovery while a mRS score of 3–6 at 90 days indicates poor functional recovery [28]. Only 11.1–28.0% of patients in the included case series and cohort studies had a modified rankin score 2 at the 3‑month follow-up, suggesting that most patients did not regain functional independence post MT for CCE. In Bruggeman et al. [21]’s study, 29% (15/51) of CCE patients achieved functional independence (mRS score of 0–2), compared to 41% (1159/2823) of non-CCE patients (*p* = 0.009). However, after propensity matching, the presence of CCE was not significantly associated with worse functional outcomes [21]. The four studies which reported good functional outcomes were all case reports [11, 19, 24, 25]. Moreover, 5 of 14 studies compared NIHSS scores on admission and post-thrombectomy, with 4 describing a ≥ 3 points reduced in NIHSS [4, 11, 17, 19, 21]. The median improvement in NIHSS score ranged from a 2-point [21] to a 9-point improvement [11].

### Mortality

Only 4 of the 14 studies reported mortality rates following MT for CCE [12, 17, 18, 21]. MT for CCE was associated with a 3-month mortality rate of 0–62.5% across these studies. Koh et al. did not report any mortality (in hospital or 90-day) in their retrospective case series of 5 patients [17]. Dobrocky et al. reported a 3-month mortality rate of 62.5% (5/8 patients) [18]. Similarly, Maurer et al. reported a 90-day mortality rate of 55.9% following MT for CCE (*n* = 19/34) [12]. Bruggeman et al. reported a 90-day mortality rate of 35% for CCE patients, and 29% for non-CCE patients [21]. There were no deaths reported during or immediately following MT in any study.

### Adverse Effects

6 of 14 included studies reported adverse effects following MT [4, 12, 18, 20, 21, 26]. The most common adverse effects were haemorrhagic complications, which was observed even among patients who had not received IV tissue plasminogen activator (TPA). Other complications included detachment of the stent retriever, thrombus embolisation, vessel perforation, pneumonia, stroke progression and recurrent stroke.

## Discussion

Early recanalisation following LVO is associated with superior clinical outcomes following AIS [2, 29]. Large randomised controlled trials (RCTs) report that successful recanalisation rates following MT range from 59% in the MR CLEAN study to 88% in the SWIFT prime cohort [2, 30]. However, several factors may impede successful recanalisation, including vascular tortuosity, location of the occlusion, and thrombus composition [19]. AIS due to calcific emboli is a rare entity in patients undergoing thrombectomy, but is associated with worse angiographic outcomes and mortality [12]. With an increasing number of patients undergoing minimally invasive cardiac interventions, the prevalence of CCE may not only be under-estimated, but may rise in the future. In this context, our systematic review aimed to evaluate the current evidence regarding the efficacy and safety of MT in the management of patients with AIS secondary to CCE.

Two reviews have previously evaluated the role of MT in AIS secondary to CCE. Raghib et al. [10] completed a literature review in addition to presenting three case reports [10]. However, this review was not conducted systematically, limiting its reliability and introducing potential selection bias in study inclusion. Similarly, Grand et al. [4] conducted a meta-analysis on this topic (*n* = 136); however, their search was limited to between January 2015 and March 2020 [4]. Thus, our systematic review expands upon existing literature by evaluating six additional relevant studies published between 2020–2024.

Only 5 of the 14 included studies reported that MT was effective in the management of AIS secondary to CCE [11, 20, 21, 23, 25]. Several case reports support the role of MT in CCE management, including studies by Murakami et al. [20], Bres bullrich et al. [11], Yokochi et al. [25] and Vishwanath et al. [23]. Although, there studies may have been impacted by the small study effect and publication bias. Bruggeman et al. [21]’s prospective study concluded that successful recanalisation was achieved significantly less often in CCE patients, compared to AIS patients without CCE [21]. However, despite worse recanalisation rates, there was no significant difference in functional outcomes or mortality between groups, suggesting recanalisation alone may not predict functional recovery in CCE.

In contrast, 9 of the 14 included studies reported poor recanalisation rates and functional outcomes when MT was used in the management of AIS secondary to CCE [4, 12, 16–19, 22, 24, 26]. Koh et al. [17]’s case series concluded that MAT was unable to remove CCE in all 5 included patients with AIS. The two patients who experienced favourable functional outcomes (mRS score < 2) at 3 months were those with good collateral cerebral blood flow [17]. Dobrocky et al. [18]’s retrospective case series concluded that only 12.5% of CCE patients achieved a TICI 2 b–3 score and a MRS of 0–2 at 3 months. In their study, 62.5% of patients died by the 3‑month follow-up mark and one was left severely disabled (mRS 5) [18]. Similarly, Maurer et al. [12]’s retrospective study determined that an excellent functional outcome (mRS 0–1) was achieved in only 20.6% of CCE patients post-MT and functional independence (mRS 0–2) in only 26.5%. Maurer et al. [12] also reported a 90-day mortality rate of 55.9% [12]. A multi-centre retrospective study by Grand et al. [4] similarly reported successful recanalisation in only 57% of cases and good clinical outcomes at 3‑month follow-up for 28% of patients [4]. Case studies by Schirmer et al. [16], Kwak and Park [19], Mosqueira et al. [22], Azad et al. [24] and Chiaroni et al. [26] all attempted to remove a CCE via MT, but were unsuccessful [16, 19, 22, 24, 26]. Haemorrhagic complications were the most common adverse effect associated with MT.

Histopathological, clinical and patient factors may partly explain the poor efficacy of MT in the management of AIS secondary to CCE. First, calcified clots contain significant amounts of calcium phosphate, which may influence their mechanical properties and increase the procedural difficulty of MT [11]. Second, as CCE is a rare cause of AIS, therapeutic experience is lacking which may contribute to inconsistent findings [10]. Third, patients with calcified emboli are more likely to have other risk factors that correlate with poor functional outcomes and mortality, such as significant atherosclerotic and cardiac disease that may confound the findings [18].

Although no study directly compared the effectiveness of different thrombectomy techniques, several reported their chosen techniques and outcomes. A penumbra reperfusion catheter and continuous manual aspiration was employed in Koh et al. [17]’s case series; however, recanalisation was not achieved for any participant (*n* = 5). Koh et al. postulate that this technique failed due to CCE’s hardened features and protrusion into vessel lumen [17]. Kwak & Park posit that the harder consistency of CCE may make it difficult to place the catheter tip into the calcific embolus in order to establish the vacuum necessary for manual aspiration [19]. Bruggeman et al. [21]’s study found that stent retrievers were more frequently used as the primary treatment device and were significantly associated with successful recanalization in CCE patients (*p* = 0.04). However, due to the small sample size, this study was unable to draw definitive conclusions from this finding [21]. Contrastingly, Dobrocky et al. [18], only achieved 12.5% recanalisation rates using the stent retriever. As a stent retriever works by compressing and entrapping thrombus material into the stent struts, the high resistance of CCE to deformation may impair the effectiveness of the device [18]. Maurer et al. [12] and Grand et al. [4] achieved higher recanalisation rates of 57.5% and 57% respectively. Interestingly, a combination approach involving both a stent retriever and an aspiration catheter was the most common treatment modality in both studies [4, 12]. This is supported by an *in vitro *study by Johnson et al., which showed that a combined technique of stent retrieval and manual aspiration using a balloon guided cannula was the most effective approach to remove CCE [31].

There were several significant limitations across the included studies. First, all but one study [21] lacked a control or comparison group, and therefore recanalisation rates and functional outcomes could only be compared to other published cohorts. As patients with CCE are more likely to be older and possess other comorbidities, including cardiac disease and atherosclerosis, direct comparison with other cohorts may not be valid and may introduce bias due to these differences. For example, in Maurer et al. [12]’s study the mean age was 78 years, compared to 68 years in the Trevo registry and HERMES data [32, 33]. This age difference may partly explain the dismal mortality rate of 55.9% at 90 days in their cohort, compared with the 13.9% and 15.3% mortality rate reported by the Trevo registry and HERMES collaborators respectively. Although, Slater et al. [34] suggests that the magnitude of benefit following MT is highest for those aged above 70 [34]. Bruggeman et al. [21] conducted a 1‑1 propensity score matching analysis, adjusting for baseline variables between the CCE and non-CCE group. In this context, Bruggeman et al. [21] found that the presence of CCE was not significantly associated with worse functional outcomes [21]. However, patient comorbidities alone fail to explain the substantially lower recanalisation rates when compared to the Trevo registry and HERMES cohorts, suggesting thrombus composition and device resistance may contribute.

Very few studies provided histological evidence to confirm the imaging diagnosis of CCE, which raises concern of the diagnostic accuracy based solely on imaging characteristics. This may have led to an underestimation of CCE (false negative), or the erroneous inclusion of non-CCE in the dataset (false positive). This may have led to non-differential misclassification that may bias effect estimates towards the null. In Bruggeman et al. [21]’s study, for example, CCE was confirmed on CT by an observer who received only weeks of training in stroke CT interpretation, rather than by a neuro-radiologist, which limits the internal validity of the study. In this study, three thrombi of CCE patients were analysed for the presence of calcium, which was only confirmed in one sample [21]. Moreover, the Grand et al. [4] study was conducted in France where magnetic resonance remains the first line imaging modality [4]. However, MRI is less sensitive at diagnosing CCE, and therefore CCE may have been underestimated in this study.

Regarding the assessment of bias, the Newcastle Ottawa Scale was used to evaluate for bias in the included cohort studies, while the MURAD tool was used to assess bias in case reports and case series. Selection of the non-exposed cohort was poor, as no information was presented on patients with CCE and LVO not undergoing MT or undergoing other therapies such as IV thrombolysis. Thus, the relative harms or benefits of MT could not be determined. Moreover, all studies had a small sample size, with the largest study including only 55 patients [21]. This limitation is difficult to mitigate in future studies, given the rarity of CCE. A multi-centred approach with a long duration for participant inclusion is therefore recommended. Furthermore, most studies included were case reports or series, and only one study [21] was prospective in nature. The reliance on retrospective studies increases the likelihood of selection and recall bias.

This review’s limitations are consistent with those of the included studies. That is, the majority of the evidence is low-level with small sample sizes. Publication bias may have also impacted this review. Conference abstracts and non-English texts were also excluded.

Further research is required to clarify the efficacy and safety of MT in the management of AIS secondary to CCE. Future projects should focus on comparing the effectiveness of different MT techniques. Provided the limitations associated with the stent retriever in previous studies, alternative techniques may also be considered, such as primary aspiration, intracranial stenting and intra-arterial rTPA. These techniques may be first trialled on pre-clinical models. Large randomised controlled trials, with longer follow-up durations and larger sample sizes, are necessary. Retrospective and prospective cohort studies may also be beneficial, but they should include propensity-matching for covariates including age and cardiovascular disease.

## Conclusions

9 of the 14 included studies reported poor recanalisation rates (less than 57.5%) and functional outcomes when mechanical thrombectomy was used in the management of AIS secondary to CCE. This was often accompanied by a high rate of mortality at 3‑month follow-up. Evidence suggesting good outcomes mainly stems from individual case reports, which may be prone to publication bias. One prospective study concluded that while patients with AIS and CCE experienced lower rates of recanalisation than stroke patients without CCE, after propensity matching, there was no significant difference in functional outcomes or mortality. Some reports observed higher rates of recanalisation with combined techniques. However, as no study directly compared the effectiveness of different MT techniques, comparative efficacy remains uncertain. Overall, the available evidence is primarily low-level evidence stemming from case reports, case series and small cohort studies without comparison groups. Therefore, the conclusions are made from a low certainty of evidence and should be interpreted with caution. Further research, in the form of well-designed RCTs, with larger sample sizes, is needed to better evaluate the role of MT in this patient population.

## Supplementary Information

ESM1: Supplementary material 1
