# Supplementary material for: Mechanical Thrombectomy in the Management of Acute Ischemic Stroke Secondary to Calcified Cerebral Emboli: a Systematic Review
Source: Clin Neuroradiol. 2026 Jan 28;36(2):295–307. doi: 10.1007/s00062-025-01611-7 (PMC13319450; doi:10.1007/s00062-025-01611-7)
Supplement: Supplementary file 1 — ESM1: Supplementary material 1 [file 62_2025_1611_MOESM1_ESM.docx]

**Supplementary info 1: PRISMA research checklist**


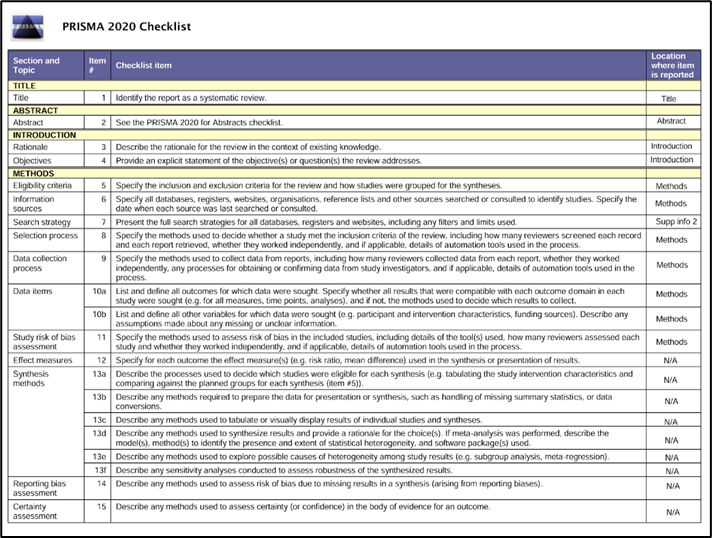


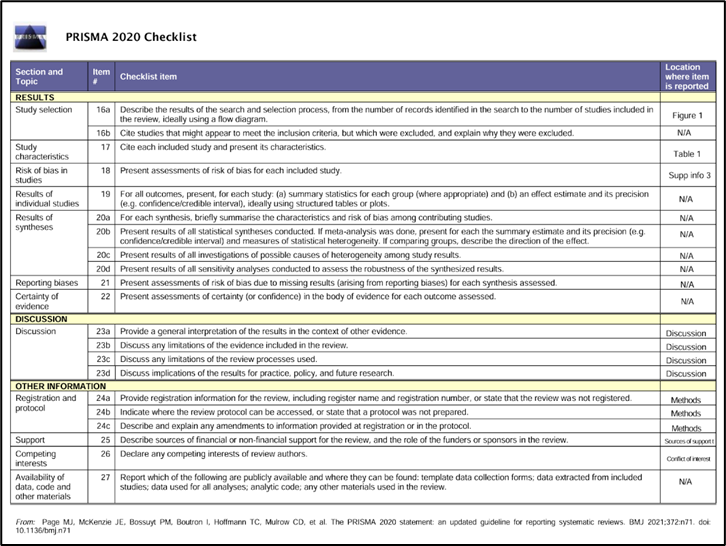


**Supplementary info 2: Search strings**

Scopus:

N/A

EMBASE:

(Calcif* and embol* and (thrombectomy or endovascular or recanalisation or retrieval)).mp. [mp=title, abstract, heading word, drug trade name, original title, device manufacturer, drug manufacturer, device trade name, keyword heading word, floating subheading word, candidate term word]

Pubmed:

"calcif*"[All Fields] AND "embol*"[All Fields] AND ("thrombectomy"[MeSH Terms] OR "thrombectomy"[All Fields] OR "thrombectomies"[All Fields] OR "endovascular"[All Fields] OR ("recanalisation"[All Fields] OR "recanalisations"[All Fields] OR "recanalise"[All Fields] OR "recanalised"[All Fields] OR "recanalising"[All Fields] OR "recanalisation"[All Fields] OR "recanalisations"[All Fields] OR "recanalise"[All Fields] OR "recanalised"[All Fields] OR "recanalisers"[All Fields] OR "recanalises"[All Fields] OR "recanalising"[All Fields]) OR ("retrievability"[All Fields] OR "retrievable"[All Fields] OR "retrieval"[All Fields] OR "retrievals"[All Fields] OR "retrieve"[All Fields] OR "retrieved"[All Fields] OR "retrieves"[All Fields] OR "retrieving"[All Fields]))

Translations

thrombectomy: "thrombectomy"[MeSH Terms] OR "thrombectomy"[All Fields] OR "thrombectomies"[All Fields]

recanalisation: "recanalisation"[All Fields] OR "recanalisations"[All Fields] OR "recanalise"[All Fields] OR "recanalised"[All Fields] OR "recanalising"[All Fields] OR "recanalisation"[All Fields] OR "recanalisations"[All Fields] OR "recanalise"[All Fields] OR "recanalised"[All Fields] OR "recanalisers"[All Fields] OR "recanalises"[All Fields] OR "recanalising"[All Fields]

retrieval: "retrievability"[All Fields] OR "retrievable"[All Fields] OR "retrieval"[All Fields] OR "retrievals"[All Fields] OR "retrieve"[All Fields] OR "retrieved"[All Fields] OR "retrieves"[All Fields] OR "retrieving"[All Fields]

Cochrane:

N/A

Medline:

(Calcif* and embol* and (thrombectomy or endovascular or recanalisation or retrieval)).mp. [mp=title, book title, abstract, original title, name of substance word, subject heading word, floating sub-heading word, keyword heading word, organism supplementary concept word, protocol supplementary concept word, rare disease supplementary concept word, unique identifier, synonyms, population supplementary concept word, anatomy supplementary concept word]

**Supplementary info 3: Risk of bias assessment**

Supplementary table 1: Risk of bias assessment (Newcastle Ottawa Scale for case-control and cohort studies)

|  | Selection | | | | Comparability | Outcome | | | Total score | Interpretation |
| --- | --- | --- | --- | --- | --- | --- | --- | --- | --- | --- |
| Cohort studies | Representativeness of the exposed cohort | Selection of the non-exposed cohort | Ascertainment of exposure | Demonstration that outcome of interest was not present at the start of the study | Control of important and additional factors | Assessment of outcome | Follow-up long enough for outcomes to occur | Adequacy of follow-up of cohorts |  |  |
| Maurer (2020) | 1 | 1 | 1 | 0 | 0 | 1 | 1 | 1 | 6 | Moderate quality |
| Bruggeman (2021) | 1 | 1 | 1 | 1 | 1 | 1 | 1 | 1 | 8 | Good quality |
| Grand (2022) | 1 | 0 | 1 | 1 | 1 | 1 | 1 | 1 | 7 | Good quality |

Supplementary table 2: Risk of bias assessment (MURAD tool for case studies and case series)

|  | Patient selection | Ascertainment | Casuality | Reporting | Interpretation |
| --- | --- | --- | --- | --- | --- |
| Schirmer (2008) | The selection method of the patient is unclear but the chosen patient satisfies the criteria of having AIS due to CCE. | The exposure is adequately ascertained. The outcomes are well defined by post-thrombectomy imaging. | Antiplatelets were administered and optimally titrated before thrombectomy. The timing of antiplatelet administration may have contributed to the residual stenosis of the occluded vessel.  Follow up was imaging, ranging from 2 days to 1 year. | The case is described in reasonable detail in terms of imaging changes after thrombectomy. However, more details on functional outcome could be included in order to allow practitioners to make inferences related to their own practice. | Medium quality |
| Koh (2017) | 5 patients are selected based on CT and cerebral angiography, identifying CCE as the cause of AIS. | The exposure is ascertained, with a focus on assessing functional outcomes. However, MT failed to remove CCE in all patients, so the outcomes might not be relevant to the effectiveness of MT. | The authors explored causes that could have contributed to the observed outcome. The failure of MT was mainly due to the hard feature of calcified plaque and the   migration of calcified plaques due to complications of calcified vascular diseases. Follow-up period was sufficient, ranging from discharge time to 3 months for NIHSS and mRS scores respectively. | The case is described in reasonable detail to allow other investigators to replicate the research or make inferences related to their own practice.  Details such as the location of each patient’s potential embolic source and emboli characteristics were included. | High quality |
| Dobrocky (2018) | 8 patients are chosen based on having sudden onset of neurological symptoms without pre-existing transient neurological attacks and satisfy the inclusion criteria, which is assessing the effectiveness of MT in AIS patients due to calcified intracranial thrombus. | The exposure is adequately ascertained. The outcomes are well defined, with a focus on post-thrombectomy TICI score, functional outcomes and mortality rate. | The authors mentioned causes that could have contributed to the observed outcome, which was low recanalisation rates in a majority of patients. Factors such as thrombus characteristics, generalized atherosclerosis, cardiac disease and old age can contribute to the poor outcomes after thrombectomy. Follow-up period was adequate, with an mRS score at 3 months. 1 patient was lost to follow up. | The case is described in reasonable detail to allow other investigators to replicate the research or make inferences related to their own practice. Each patient’s recanalisation rate, functional outcome and thrombectomy compilations are included. | High quality |
| Kwak (2018) | The selection method of the patient is unclear but the chosen patient satisfies the criteria of having AIS due to CCE. | The exposure is adequately ascertained. The outcomes are well defined by comparing NIHSS and mRS scores before and after thrombectomy. However, recanalisation rates were not reported. | The authors did not explicitly mention factors that could have contributed to the failure of CCE retrieval by aspiration thrombectomy. However, the features of the ERIC device resulting in successful recanalisation was explored. Follow-up period was adequate, with an mRS score at 3 months. | The case is described in reasonable detail in terms of assessing functional outcomes after thrombectomy. However, more details on recanalisation rate could be included, in order to measure the effectiveness of thrombectomy on treating CCE and to allow practitioners to make inferences related to their own practice. | Medium quality |
| Murakami (2019) | The selection method of the patient is unclear but the chosen patient satisfies the criteria of having AIS due to CCE. | The exposure is adequately ascertained. The outcomes are well defined by post-thrombectomy imaging, TICI and mRS scores. However, the outcomes are predominantly focused on neuromonitoring methods during TAVI procedure. | The authors mentioned causes that could have contributed to the observed outcome of successful recanalisation after TAVI, for instance the histopathological features of emboli.  Post-thrombectomy haemorrhagic complications were reported and continuous heparin administration, which started in the TAVI procedure, was stopped. Follow-up period was an mRS score at 30 days, which could be longer. | The case is described in reasonable detail in terms of recanalisation rate and functional outcomes after thrombectomy. However, a majority of details focus on neuromonitoring and the use of local or general anesthesia during TAVI. Additional details, such as how TAVI leads to the complication of CCE-related AIS and the potential causes of CCE could be included in order to allow practitioners to make inferences related to their own practice. | Low quality |
| Bullrich (2021) | The selection method of the patient is unclear but the chosen patient satisfies the criteria of having AIS due to CCE. | The exposure is adequately ascertained. The outcomes are well defined, with a focus on post-thrombectomy TICI, NIHSS and mRS scores. | The authors explored causes that could have contributed to the observed outcome, which was successful recanalisation. Factors such as the nature, potential source of CCE and implications of the patient’s condition were mentioned. Follow-up period was NIHSS and mRS scores 48 hours post-thrombectomy, which could be longer. | The case is described in reasonable detail to allow other investigators to replicate the research or make inferences related to their own practice. | High quality |
| Mosqueira (2022) | The patient selection method is unclear, but the selected patients satisfy the study’s area of focus, which is looking at the outcomes of patients who presented with AIS secondary to CCE. | Exposure was ascertained in the sense that one patient underwent thrombectomy, however the direct outcomes of that patient are not fully discussed, and it is broadly reported that MT was not successful. | Although the authors did not investigate other causes for the patients’ outcomes, AIS due to CCE can be considered as the main reason for their situation based on their presentation and imaging findings, and other reasons can be reasonably excluded. Follow up was standard at 3 months for mRS. | More details could have been included with regards to the interventions received by each patient and the respective outcomes. There is sufficient details to allow others to replicate this study, however it cannot be used for decision making at practices as it does not comment on specific outcomes. | Low quality |
| Vishwanath (2023) | The patient selection method is unclear but the patient does satisfy the field of the report, which is AIS due to CCE. | Exposure is adequately ascertained as the patient went through MT. The outcomes are also adequately ascertained by mentioning the TICI score post thrombectomy. | The authors investigated the underlying cause of the embolus and appear confident about the source of the embolus and that the CCE was the reason behind the presentation. | The mentioned case is described in reasonable detail to allow other researchers to replicate the research, however more details could have been covered. Use in decision making is limited due to the absence of some details such as delta NIHSS. | Medium quality |
| Azad (2023) | Patient selection method is unclear but the patient satisfies the topic covered by the study which is AIS secondary to a CCE from pelvic phlebolith. | Exposure is adequately ascertained and the outcomes are also clearly stated and ascertained. | The authors investigated the underlying cause of the embolus and appear confident about the source of the embolus and that the CCE was the reason behind the presentation. | The mentioned case is described in reasonable detail to allow other researchers to replicate the research or make inferences related to their own practice. | High quality |
| Yokochi (2024) | The patient selection method is unclear but the patient does satisfy the field of the report, which is AIS due to CCE. | Exposure is adequately ascertained. The outcomes are also clearly ascertained and mentioned. | The authors successfully carried out investigations to understand the underlying cause of the presentation and causality is confirmed. | The mentioned case is described in reasonable detail to allow other researchers to replicate the research or make inferences related to their own practice, however additional details (such as post procedure NIHSS) are missing. | High quality |
| Chiaroni (2024) | The patient selection method is unclear but the patient does satisfy the field of the report, which is AIS due to CCE. | Exposure is adequately ascertained. Outcomes are mentioned qualitatively and the report lacks post intervention NIHSS and mRS data. | The authors successfully carried out investigations to understand the underlying cause of the presentation and causality is confirmed due to the presenting nature of the condition. | The mentioned case is described in reasonable detail to allow other researchers to replicate the research. Other researchers may find it difficult to make inferences related to their own practice as additional details (such as post procedure NIHSS and mRS) are missing. | Medium quality |
